# A case of adrenal lymphangioma successfully treated with laparoscopic partial adrenalectomy

**DOI:** 10.1002/iju5.12618

**Published:** 2023-09-28

**Authors:** Heisuke Iijima, Taisho Noda, Kosuke Uchida, Yasuhiro Hakamata, Akira Fujisaki, Shin Imai, Yoshiro Otsuki, Tatsuaki Yoneda

**Affiliations:** ^1^ Department of Urology Seirei Hamamatsu General Hospital Shizuoka Japan; ^2^ Department of Pathology Seirei Hamamatsu General Hospital Shizuoka Japan

**Keywords:** adrenal lymphangioma, adrenal tumors, incidentaloma, laparoscopic surgery, lymphangioma

## Abstract

**Introduction:**

Adrenal lymphangioma is a rare benign tumor of lymphatic origin, usually incidentally detected from various imaging studies taken for an unrelated purpose. We present a case of a right adrenal lymphangioma treated successfully with surgical intervention.

**Case presentation:**

A 36‐year‐old previously healthy woman was referred to our urology department for a right adrenal mass, discovered during a routine health checkup. The tumor had no endocrinological activity, and the patient opted for surgical resection following a concern for malignancy. A laparoscopic right partial adrenalectomy was performed, and on histological examination, the tumor was diagnosed as right adrenal lymphangioma.

**Conclusion:**

Adrenal lymphangiomas lack disease specific radiological characteristics that allow for a definitive diagnosis from imaging alone. To rule out tumors of potentially malignant nature, surgical intervention should be considered.

**Abbreviations & Acronyms**
CT: computed tomography
H&E: hematoxylin and eosin
MRI: magnetic resonance imaging

**Keynote message**

Adrenal lymphangioma is a rare benign tumor of lymphatic origin. This tumor has no disease‐specific characteristics that allow for diagnosis solely from imaging studies. Here, we report a case of right adrenal lymphangioma successfully treated with laparoscopic partial adrenalectomy.

## Introduction

Adrenal lymphangioma is a rare benign tumor of lymphatic origin. Whereas most lymphangiomas are found during infancy, usually in the cranial region, adrenal lymphangiomas are mostly detected incidentally during adulthood from various imaging studies. Here, we report a case of a right adrenal lymphangioma successfully treated with laparoscopic partial adrenalectomy.

## Case presentation

**Fig. 1:**
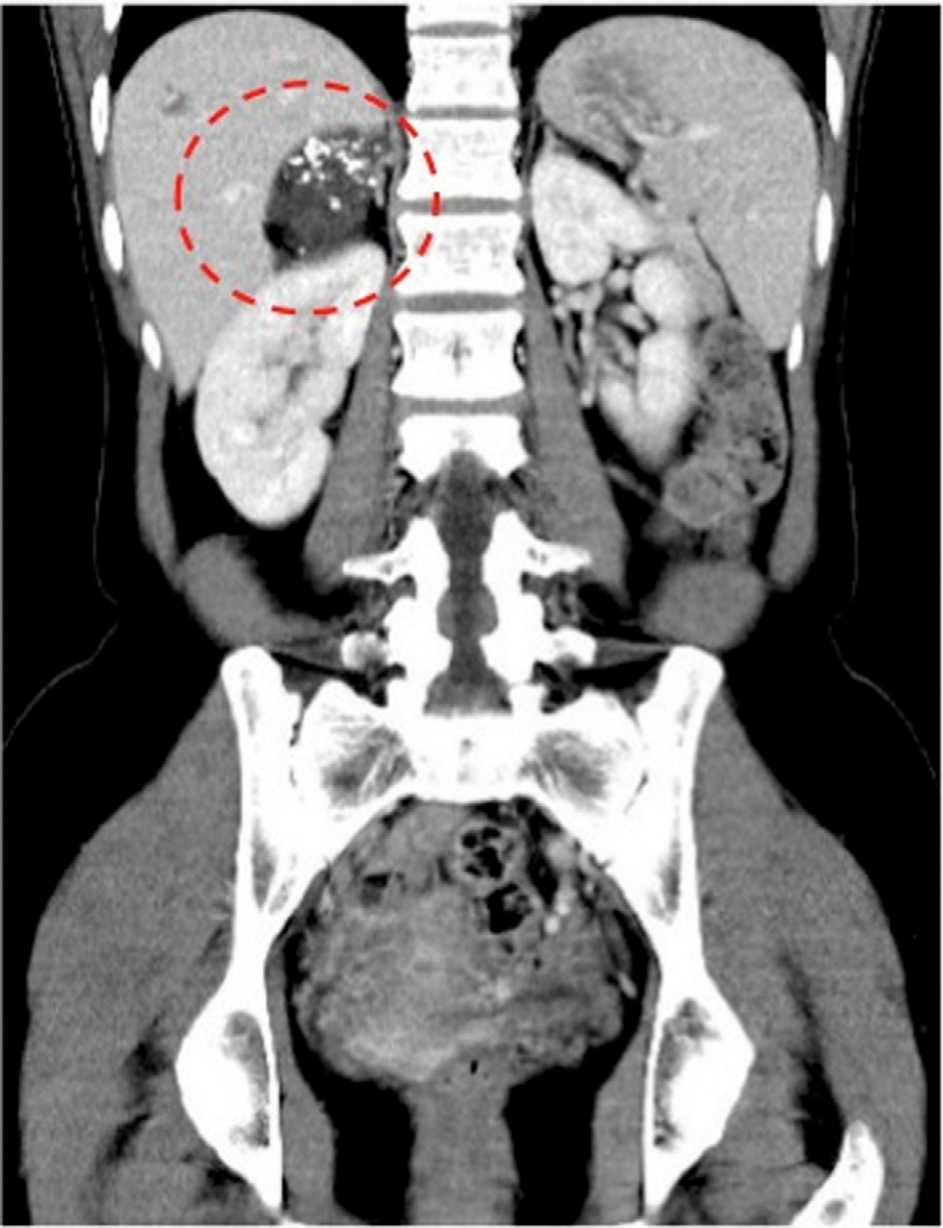
Contrast CT scan, coronal section. Right adrenal cystic tumor with calcifications shown in red dashed circle.

**Fig. 2:**
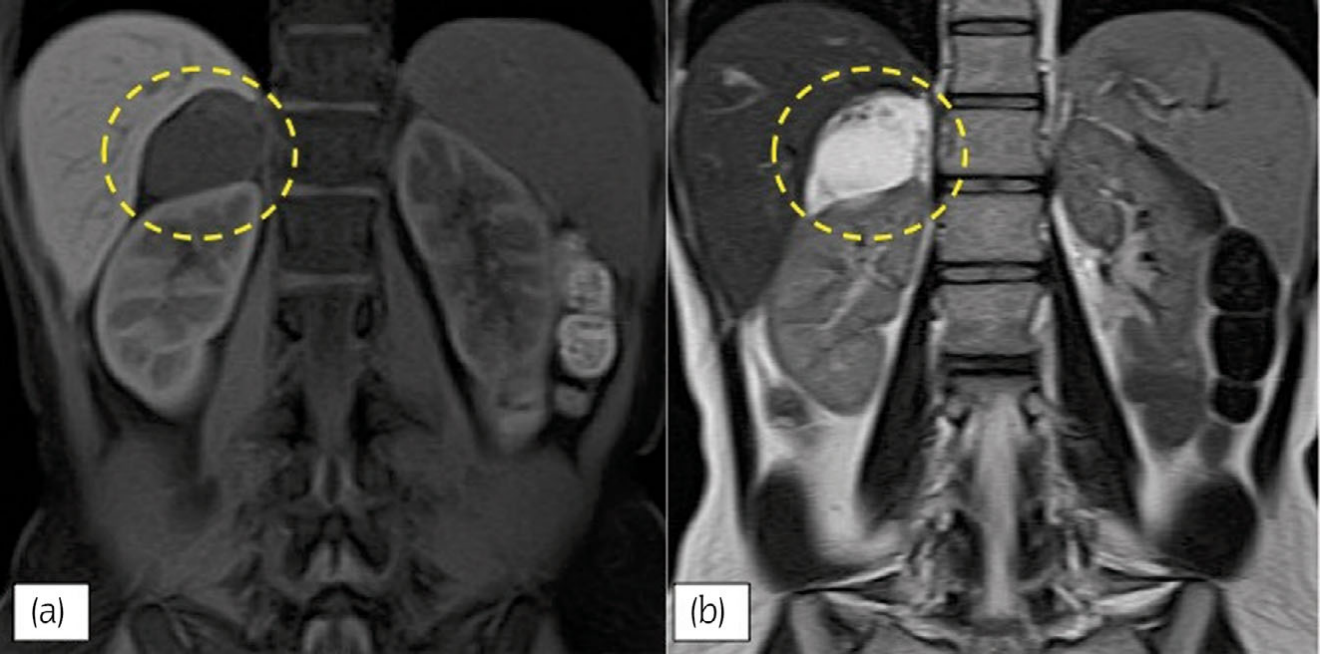
Abdominal MRI, coronal section. (a) T1 weighted image, showing hypointense adrenal tumor, encircled. (b) T1 weighted image, showing hyperintense adrenal tumor, encircled.

A 36‐year‐old previously healthy woman was referred to our hospital for a right cystic adrenal mass, which was discovered incidentally during her routine medical checkup. A contrast CT scan showed a poorly enhanced 5 cm solitary mass on the right adrenal gland, with a polycystic appearance with some calcification (Fig. 1). On MRI, the mass was uniformly hypointense on T1 weighted image (Fig. 2a), and hyperintense on the T2 weighted image (Fig. 2b). An endocrinological workup was performed prior to referral, with no detectable abnormalities, suggesting a non‐functioning adrenal mass.

The patient opted for surgery from her concerns about a possible malignancy, or future hemorrhage.

The surgery was performed under general anesthesia in the left semi‐lateral decubitus position. A 12 mm laparoscopic port was positioned slightly lateral from the right midclavicular line, 3 cm caudal from the ribcage. A 5 mm port was placed on the same axial plane, on the right midclavicular line for the right‐hand access, and on the anterior axillary line for the left hand. A 5 mm assistant port was placed caudally from the xyphoid.

**Fig. 3:**
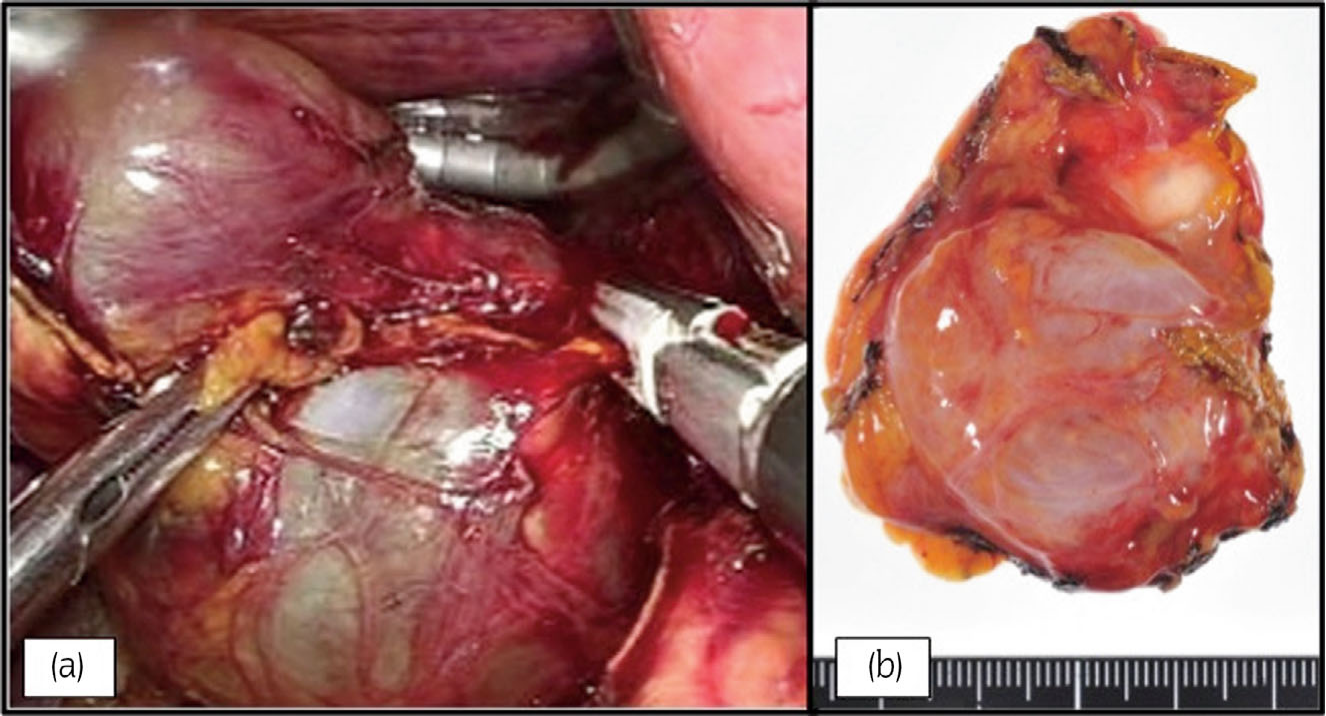
Laparoscopic adrenalectomy. (a) Cystic adrenal tumor captured during surgery. (b) Macroscopic image of resected tumor.

The tumor had a round, polycystic appearance, with clear visible margins with the adrenal gland (Fig. 3a,b). The lack of adhesions between the tumor and surrounding tissue was strongly suggestive of a benign tumor, and an intraoperative decision for a partial adrenalectomy was made. A complete resection of the tumor was performed with much of the functioning adrenal gland left intact. The operation time was 1 h 53 min, with 5 mL blood loss, and the patient was discharged 4 days post‐surgery. On cross‐section, the tumor was polycystic, containing a clear, serous substance.

**Fig. 4:**
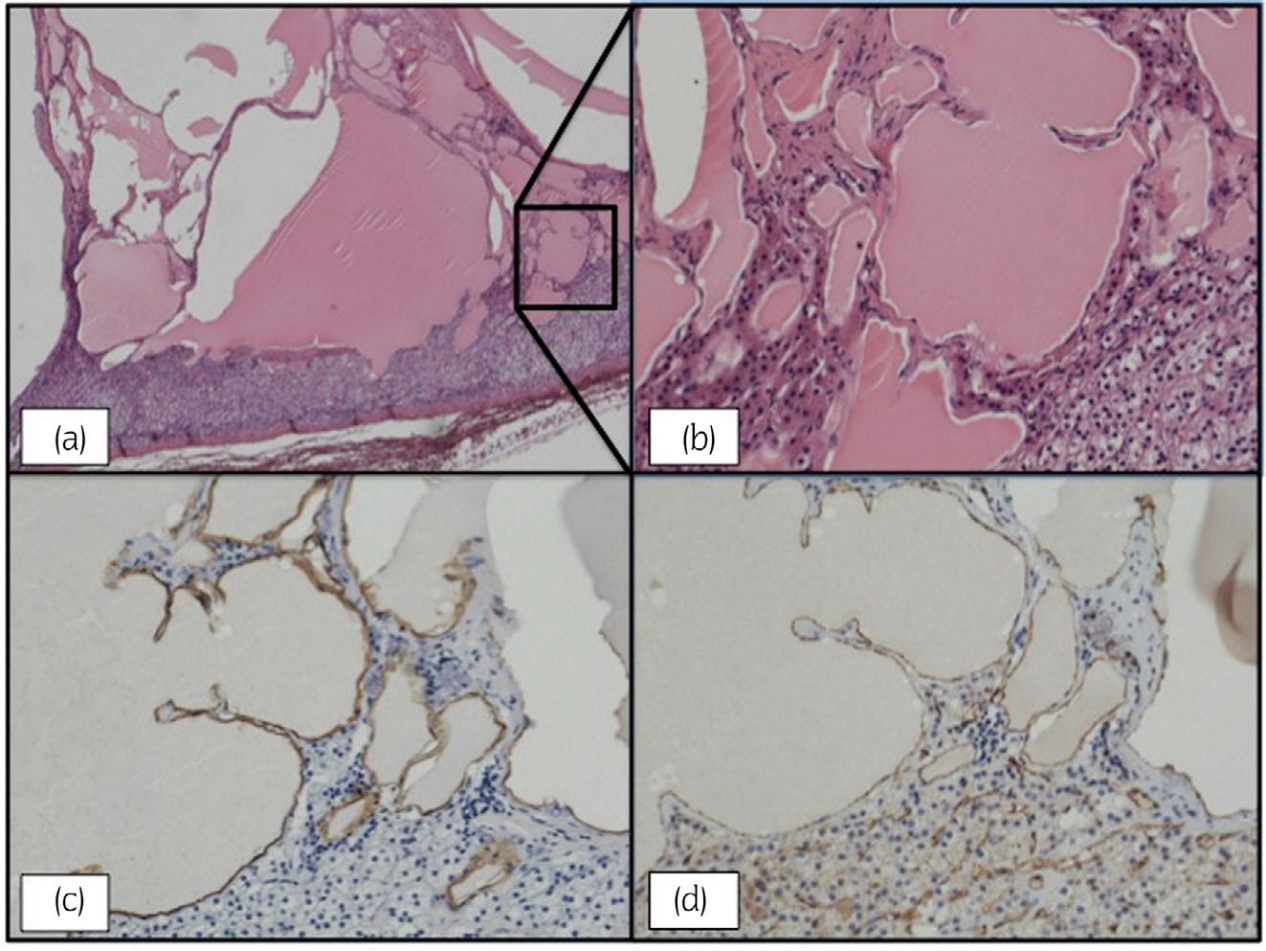
Resected adrenal tumor. (a) H&E stain, ×30 magnification. Cystic endothelial walls with calcification (shown in pink). (b) H&E stain, ×150 magnification, calcification (in pink), with normal adrenal tissue (right). (c) Immunohistochemistry stained with D2–40. (d) Immunohistochemistry, stained with CD31.

Histologically, on H&E staining, fibrotic cysts containing calcifications were observed, with clear borders with the normal adrenal tissue (Fig. 4a,b). On immunohistochemistry, the endothelial cell lining of the cyst wall was stained positive for D2–41 and CD31, a phenotype characteristic of lymphatic tissue, together suggestive of the final diagnosis of adrenal lymphangioma (Fig. 4c,d).

## Discussion

Lymphangiomas are benign tumors originating from the lymphatic system, majority of which are found during infancy, commonly in the cranial and axillary regions.^1^ Lymphangiomas found during adulthood are relatively rare, usually discovered incidentally as an intraabdominal or retroperitoneal mass during routine medical checkups or imaging for other unrelated purposes.^2^, ^3^

Adrenal lymphangiomas are therefore a rare occurrence, although there is an increase in its reported incidence, presumably owing to the advancement and availability of various imaging modalities.^4^ To the best of our knowledge, fewer than 70 cases have been reported in the English literature. Its precise mechanism of pathogenesis is unknown, although causes such as congenital abnormalities of lymphatic drainage and lymphatic congestions due to trauma or inflammations have been hypothesized.^1^ Most lymphangiomas are asymptomatic, although incidences where these tumors were discovered after symptoms such as abdominal pain, palpable abdominal mass, hemorrhage, and infections have been reported.^1^, ^2^ Some have also reported adrenal lymphangiomas accompanied by abnormal hormonal secretions, presumably due to compression of the adrenal artery or medulla.^5^ Ellis *et al*. observed that these tumors are more frequently found in women, and more commonly seen in the right adrenal gland.^1^

As it was the case for this patient, on a histological level, lymphangiomas are often seen on H&E staining as polycystic lesions lined with endothelium‐like cells.^1^, ^2^, ^4^

Immunohistochemistry is also a very useful tool to confirm the tumor's lymphatic origin. We utilized CD31 and D2‐40, which are specific markers used to detect tissues of lymphatic linage. Other useful markers may include CD34, factor VIII‐related antigen, and PROX1.^1^, ^2^, ^6^

Lymphangiomas lack specific radiological characteristics that allow for differentiation on imaging alone.^1^ Other cystic adrenal lesions with a similar radiological appearance that would require consideration include adrenocortical adenoma or carcinoma, pheochromocytoma, myelolipoma, neuroblastoma, schwannomas, and ganglioneuromas.^1^, ^2^, ^4^

Surgical excision may be the management of choice for symptomatic or large adrenal lymphangiomas.^1^, ^2^, ^4^ Percutaneous aspiration of the cysts may be useful for diagnosis, though not for therapeutic purposes, since it is likely to recur.^1^ In practice, the lack of specific radiological characteristics of this tumor means that surgical excision serves both a therapeutic and diagnostic purpose.

Due to its rarity, the precise malignancy potential of a cystic adrenal mass is not well established, and few retrospective case series have been published. Chien reported 7 out of the 25 surgically treated cystic adrenal lesions were associated with a neoplasm (two pheochromocytomas, and single cases of neuroblastoma, myelolipoma, cortical adenoma, schwannoma, and cortical carcinoma), of which two were malignant.^7^ In addition, there is evidence to suggest that pre‐surgical diagnoses of these lesions may often be inaccurate. In cases of large‐size cysts (20 cm <), the accuracy rate may be as low as 14.8%.^4^ Considering data from these reports, a diagnostic excision of these tumors seems reasonable in medical practice.

The prognosis of adrenal lymphangiomas post‐surgery seems to be extremely good, as we were unable to find cases that reported post‐surgical relapse.

## Conclusion

We report a case of a cystic adrenal lymphangioma successfully treated with laparoscopic partial adrenalectomy. This rare and benign tumor of lymphatic origin should be included in the differential diagnoses of adrenal tumors. The potential malignant nature of a tumor should always be considered during the assessment of adrenal cystic tumors.

## Author contributions

Heisuke Iijima: Writing – original draft. Taisho Noda: Validation. Kosuke Uchida: Validation. Yasuhiro Hakamata: Validation. Akira Fujisaki: Validation. Shin Imai: Validation. Yoshiro Otsuki: Validation. Tatsuaki Yoneda: Conceptualization; supervision; writing – review and editing.

## Conflict of interest

The authors declare no conflict of interest.

## Informed consent

Not applicable.

## Approval of the research protocol by an Institutional Review Board

Not applicable.

## Registry and the Registration No of the study/trial

Not applicable.

## References

[1] Joliat G‐R , Melloul E , Djafarrian R *et al*. Cystic lymphangioma of the adrenal gland: report of a case and review of the literature. World J. Surg. Oncol. 2015; 13: 58. 25889625 10.1186/s12957-015-0490-0 PMC4335415

[2] Zhao M , Gu Q , Li C , Yu J , Qi H . Cystic lymphangioma of adrenal gland: a clinicopathological study of 3 cases and review of literature. Int. J. Clin. Exp. Pathol. 2014; 7: 5051–5056. 25197378 PMC4152068

[3] Cakir E , Aydin NE , Samdanci E *et al*. Cystic adrenal lymphangioma—report of two cases and review of the literature. J. Pak. Med. Assoc. 2012; 62: 962–964. 23139985

[4] Furihata M , Iida Y , Furihata T , Ito E . A Giant lymphatic cyst of the adrenal gland: report of a rare case and review of the literature. Int. Surg. 2015; 100: 2–8. 25594633 10.9738/INTSURG-D-14-00125.1 PMC4301289

[5] Goel D , Enny L , Rana C *et al*. Cystic adrenal lesions: a report of five cases. Cancer Rep. (Hoboken) 2021; 4: e1314. 33295135 10.1002/cnr2.1314 PMC7941571

[6] Kim KH , Lee JI , Bae JM . Significant growth of adrenal lymphangioma: a case report and review of the literature. Int. J. Surg. Case Rep. 2015; 17: 48–50. 26546871 10.1016/j.ijscr.2015.10.013 PMC4701751

[7] Chien H‐P , Chang Y‐S , Hsu P‐S *et al*. Adrenal cystic lesions: a clinicopathological analysis of 25 cases with proposed histogenesis and review of the literature. Endocr. Pathol. 2008; 19: 274–281. 18972224 10.1007/s12022-008-9046-y

